# Studies on the correlation between mutation and integration of HBV in hepatocellular carcinoma

**DOI:** 10.1042/BSR20201988

**Published:** 2020-08-21

**Authors:** Xiaofang Cui, Wei Wei, Chao Wang, Yanwei Qi, Xiao Qin, Lizhen Huang, Weiyang Li

**Affiliations:** 1 Jining Medical University, Jining, Shandong 272067, China; 2 Shandong Key Laboratory of Behavioral Medicine, School of Mental Health, Jining Medical University, Jining, Shandong 272067, China; 3 Collaborative Innovation Center for Birth Defect Research and Transformation of Shandong Province, Jining Medical University, Jining, Shandong 272067, China; 4 Lupus Research Institute, Affiliated Hospital of Jining Medical University, Jining Medical University, Jining, China; 5 Department of Medical Laboratory, Jining Hospital of Traditional Chinese Medicine, Jining, Shandong 272067, China; 6 Biotechnology and Health Center, City University of Hong Kong Shenzhen Research Institute, Shenzhen, 518057, China; 7 School of Biology and Biological Engineering, South China University of Technology, Guangzhou 510006, China

**Keywords:** HBV integration, HBV, HCC, SNV, TERT

## Abstract

It is well known that both the mutation and integration of the Hepatitis B virus (HBV) are of great significance in liver cancer, however, the relationship between mutation and integration is still unclear. In the present study, sequencing data from 426 previously published samples were analyzed and 5374 specific HBV mutations in cancer tissues were discovered. By comparing integrated samples and non-integrated samples, we found that the integrated samples had higher sample single nucleotide variants (SNVs) positive rates and SNV numbers, as well as higher sample frequency of SNV in the X region of the HBV genome. Samples with HBV integration in the telomerase reverse transcriptase (TERT) region showed higher SNV positive rates and numbers than samples without integration. Moreover, the SNVs (209 [T>G] and 531 [T>C; T>G]) were seen with higher frequency in samples with integration in the TERT region. Our study showed that the occurrence of viral integration events is closely related to the occurrence of SNV, and SNV in the X region should be more directly associated with viral integration. The present study provides an initial exploration of the relationship between HBV mutation and integration to help improve our understanding of the relationship between viral integration and mutation.

## Introduction

Chronic infection caused by the Hepatitis B virus (HBV) is a global public health problem, with approximately 350 million people suffering from it, among which 75% are Asians [1,2]. HBV infection is a major cause of cirrhosis and hepatocellular carcinoma (HCC). [3] Genotype is an important biological feature of HBV and different genotypes/subtypes will have different pathogenic characteristics. For example, genotype B often causes acute infection, while genotype C often leads to chronic infection, and subtype C is a key risk factor for the occurrence of HCC. Genotype-specific mutation profiles exist in different disease stages during the transformation from hepatitis to liver cancer. The mutations that occur during the HBV replication process contribute to the evolutionary characteristics of clonal selection and these mutations are closely related to the progression of hepatitis B-related diseases [4,5]. HBV carriers are 5–15-times more likely to develop cancer than non-carriers and the occurrence of HBV integration is correlated with an increased risk of cancer [6,7]. Studies have shown that HBV-associated liver cancer is often accompanied by HBV integration and the positive rate of HBV integration events detected in cancerous tissue samples can be as high as 85–90%, compared with hat found in tumor adjacent tissues which are only 30.7% [8–10]. Viral integration events can often occur alongside viral protein expression and the continuous regeneration of hepatocytes, which greatly increases the probability of cell carcinogenesis. Therefore, it is clear that viral integration events have an important influence on tumorigenesis [11].

A previous study revealed that the HBV integration rate is significantly different between tumor and normal tissue. Other recent studies have shown that carcinogenesis can be caused by viral integration in various ways, for example, HBV integration leads to the generation of viral proteins or defective viral proteins, which can interfere with the normal function of the tumor suppressor pathway and cause cancer. Additionally, the enhancer element of the virus could trigger abnormal transcriptional expression of important nearby genes or produce chimeric transcripts and proteins, resulting in cell dysfunction. Finally, the induced intracellular chromatin structural changes can lead to tumorigenesis by triggering genome instability, inducing progression, or by remote regulation [12–16].

Previous research has shown that there are multiple ‘hotspot’ areas where HBV integration can occur. According to Sung et al., the hotspot integration genes of HBV in liver cancer include telomerase reverse transcriptase (TERT), MLL4, and CCNE1, which affect expression of related genes. The occurrence of viral integration events is correlated with an increase in α fetal protein (AFP) and poor prognosis [10]. Lau et al. reported that HBV integration in the non-gene region Chr8p11.21 can promote liver cancer development by forming a novel chimeric transcript and activating the Wnt signaling pathway [17]. Clinical association analysis demonstrated that integration in this region results in poor clinical prognosis [17]. Hongli et al. found that 20% of early-onset liver cancer patients had an integration hotspot in the 8q24 region between the two oncogenes c-Myc and PVT1, and showed that viral integration events could lead expression changes of these two genes, which are involved in tumorigenesis, by comparing the characteristics of viral integration between early and late-onset liver cancer [18]. In 2016, the integration frequency and clinical relevance of the HBV virus in liver cancer were studied, confirming that the Chr5p15.33 (TERT-CLPTM1L) region is the hottest spot of HBV integration (the integration frequency in tumor samples was approximately 25%), and that integration events in this region could lead to poor prognosis [19]. Although the function of virus integration in these hotspots is unclear, it is speculated that it is closely related to the occurrence of liver cancer.

However, research on the relationship between viral integration and variation is still in its infancy. The present study used the previously published data of 426 HCC samples to further investigate the relationship between viral variation and integration, and found that the occurrence of HBV integration events is often accompanied by an increase in the positive rate and the number of single nucleotide variant (SNV). The significant regions and sites of SNV occurrence in integrated and non-integrated virus samples were determined, laying a foundation for further in-depth research on the relationship between viral mutation and integration.

## Materials and methods

### SNV of HBV in 426 HCC samples

Previously published data from 426 paired samples (HCC and paired normal sample) were used to carry out the analysis [19]. All data used in the present study have been deposited in the Sequence Read Archive (SRA) database under the accession codes SRA335342 and SRA447498, respectively. First, clean reads were obtained by removing all low quality, duplicated, and adaptor-contaminated reads. Burrows–Wheeler Aligner (BWA) was used to align clean reads on to the HBV genome (AB014381.1) [20]. The GATK tool was used to obtain SNPs from the cancer and adjacent normal tissues [21]. Raw variants were called by the GATK Haplotype Caller module. SNPs were filtered through the paired normal tissues to obtain the unique SNV in the cancer tissues.

### Detection and annotation of HBV integration sites

Analysis was performed using our previously published algorithm [22]. In brief, low quality, duplication, and adaptor contamination reads were all removed to obtain clean reads. Clean reads were mapped to the human (NCBI build 37, HG19) and HBV genomes (AB014381.1). Those reads in which paired-ends aligned to the human or HBV genomes were removed and the other chimeric paired-end reads were reserved. The joint position of the human and HBV sequences was the breakpoint for HBV integration. The annotation of integrated breakpoints was performed with ANNOVAR [19,23].

## Results

### The relationship between integration and SNV positive rate

**Figure 1:**
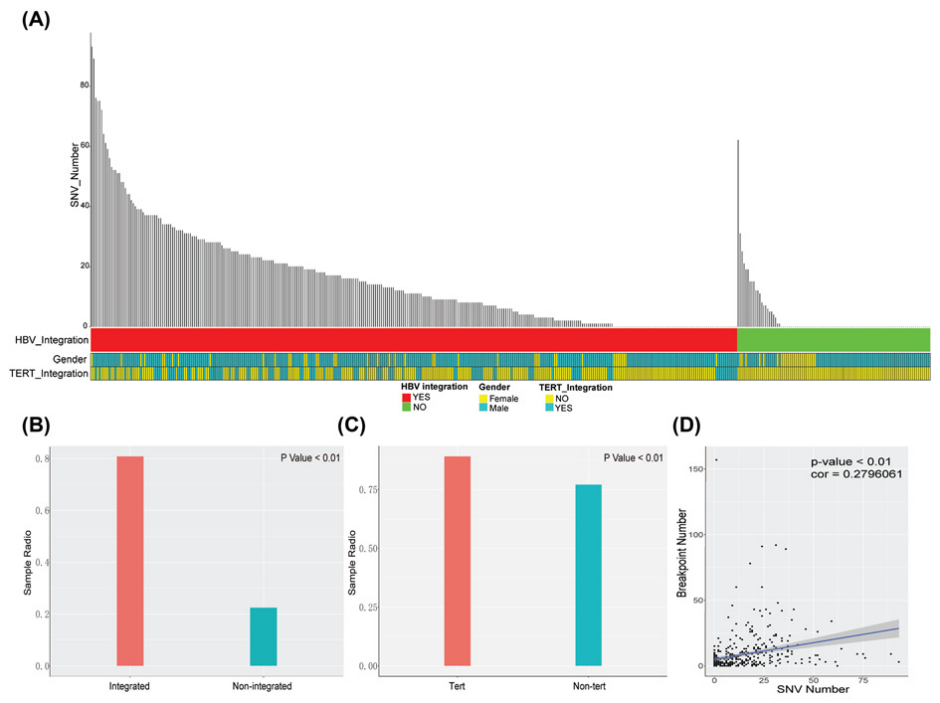
The overview of HBV integration and SNVs in 426 HCC samples (**A**) All panels are aligned with vertical tracks representing 426 individuals. The data are sorted by HBV integration, gender, TERT integration. (**B**) Integrated represents the SNV positive rate of the integrated samples, non-integrated represents the SNV positive rate of the non-integrated samples. (**C**) Non-Tert represents the SNV positive rate of the integrated samples of the non-TERT region. Tert represents the SNV positive rate of the integrated samples in the TERT region. (**D**) Represents the correlation between the number of HBV integrations (breakpoint) and SNVs.

An overview of HBV integration and SNVs is shown in Figure 1A. The positive rate of SNVs was significantly greater in the integrated samples (0.81) compared with the non-integrated samples (0.22) (Supplementary Table S1, Figure 1B, *P*<0.01) and the SNV positive rate of the integrated samples in the TERT region (0.89) was significantly different from that of the non-TERT region (0.77) (Figure 1C, *P*<0.01).

The correlation results revealed that there was significant positive correlation between the number of HBV integrations and SNVs (*P*<0.01, Cor = 0.279, Pearson correlation, Figure 1D).

### The relationship between integration and SNV quantity

**Figure 2:**
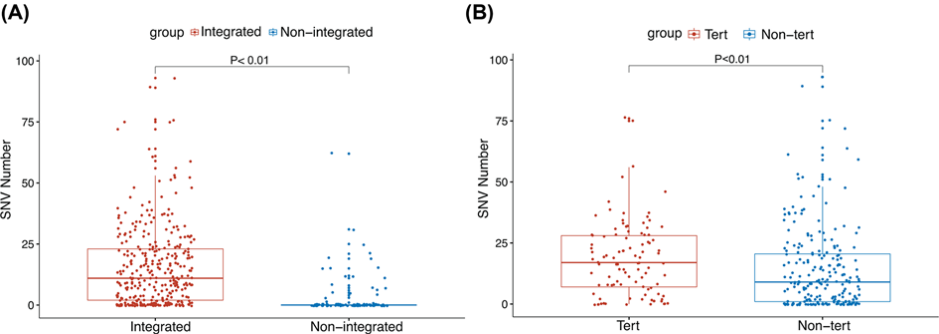
SNV quantity in different groups (**A**) Integrated represents the number of SNV in the integrated samples, non-integrated represents the number of SNV in the non-integrated samples. (**B**) Non-tert represents the number of SNV in the integrated samples of the non-TERT regions, Tert represents the number of SNV in the integrated samples of the TERT regions.

The number of SNV was significantly greater in the integrated samples than the non-integrated samples (Figure 2A, Supplementary Table S1) and the integrated samples in the TERT region had greater numbers of SNV compared with the non-integrated samples (Figure 2B, Supplementary Tables S1 and S2).

### The relationship between integration and sample SNV frequency

**Figure 3:**
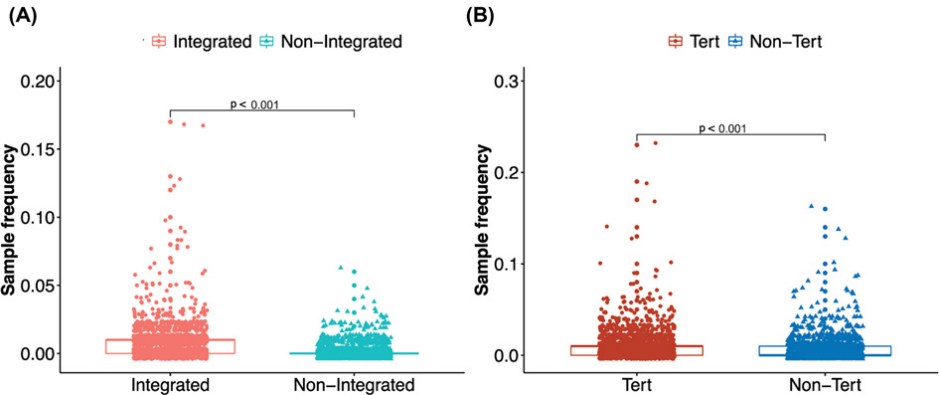
Sample frequency of SNV in different groups (**A**) Integrated represents the sample frequency of SNV in the integrated samples, non-integrated represents the sample frequency of SNV in the non-integrated samples. (**B**) Non-Tert represents the sample frequency of SNV in the integrated samples of the non-TERT regions, Tert represents the sample frequency of SNV in the integrated samples of the TERT regions.

When comparing integrated and non-integrated samples, we found that the integrated samples had a higher SNV sample frequency (Figure 3A, *P*<0.001). Comparing the samples with and without HBV integration on TERT, the results showed that the samples with HBV integration on the TERT region had a higher SNV sample frequency (Supplementary Tables S3–S5, Figure 3B, *P*<0.001).

**Figure 4:**
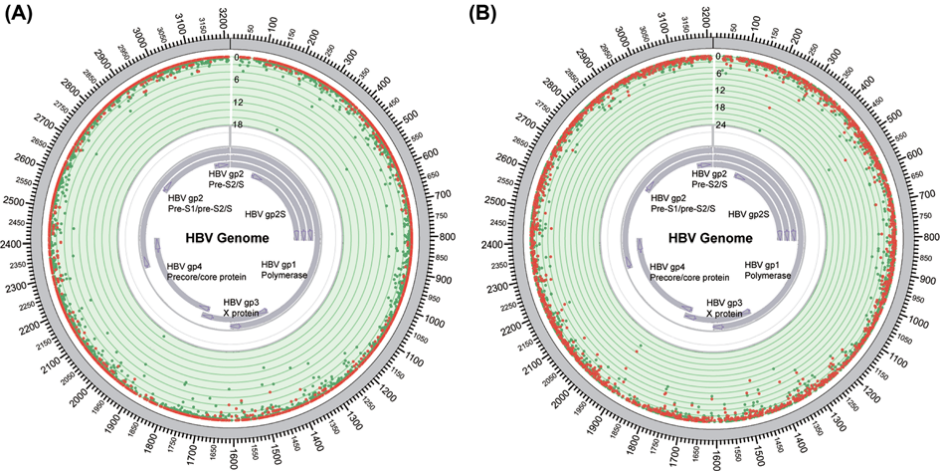
Difference in SNV frequency proportions (**A**) Red dots indicate the frequency proportion of SNV in the non-integrated samples, green dots indicate the frequency proportion in the integrated samples, the numbers represent a percentage. (**B**) Red dots indicate the SNV frequency proportion of the integrated samples in the non-TERT region, green dots indicate the proportion in the TERT region, the numbers represent a percentage.

When comparing the integrated and non-integrated samples, we found that the integrated samples had a higher frequency proportion of SNV in the X and C regions (Figure 4A, Supplementary Tables S3 and S5). Seven of the top 20 (35%) sites with significant differences were in the X region, which was a higher proportion than in the random proportion (19.8%). The frequency proportion of SNVs in the integrated samples in the TERT and Non-Tert region were significantly different in sites 209 [T>G] (Tert: 22.7%, Non-Tert: 14.1%, Figure 4B, Supplementary Table S4) and 531 [T>C; T>G] (TERT: 16.8%, Non-Tert: 10.1%, Figure 4B, Supplementary Table S4).

## Discussion

The underlying mechanism of HBV integration is still unclear. According to current studies, HBV virus integration events in liver cancer are mainly of the B/C type, which indicates that certain types of mutations are more likely to trigger viral integration events. The present study found that positive samples of viral integration have a higher positive SNV rate, and that integrated samples in the TERT region have a very high (up to 89%) SNV positive rate. The integrated samples also had a higher number of SNV. Our results indicate that HBV integration is closely related to the accumulation of viral mutations, suggesting that both factors play an important role in the occurrence of liver cancer. The TERT region has been identified as a hotspot area of HBV virus integration [10]. Considering the higher number and higher positive rate of SNV in the integrated samples in the TERT region, it can be proposed that HBV integration, accompanied with HBV mutation, is more likely to occur in this region. By comparing the SNV frequencies in the integrated and non-integrated samples, we found that the integrated samples had significantly higher sample frequency of SNV in the X and C regions, and that the differences in sample frequency were especially pronounced in the X protein region. Significant sample frequency differences in the S site were discovered in the integrated samples in the TERT and non-TERT regions, such as those at sites 209 [T>G] and 531 [T>G; T>C], indicating that viral integration is closely related to the mutations in the S regions. Since the mechanism of viral integration is not yet fully understood, it can be determined that viral integration mainly comes from double-stranded linear HBV DNA, which may be generated through the integration of microhomology-mediated end joining (MMEJ) and non-homologous end joining (NHEJ) [19,24]. There may be several mechanisms by which HBV integrations and SNVs are associated. First, certain mutations may be directly linked to HBV replication and the hepatic micro-environment, and are thus likely to generate more double-stranded linear DNA, which subsequently raises the possibility of HBV integration. Second, a number of SNVs in certain regions of the HBV genome might provide host genome integration advantages (for example, promoting MMEJ and NHEJ). Third, once HBV integration has succeeded (driven by certain HBV SNVs and/or dependent on the context of integration sites), infected cells may be endowed with distinct advantages in terms of survival and proliferation. Finally, cellular chromatin accessibility and associated chromatin modifications may act as additional layers of determinants of HBV integration. In our study, we observed positive correlations between the number of HBV integrations and SNVs which might be the results of any of these mechanisms. With respect to whether it is the number of mutations or certain key mutations that are important in HBV integration events, we speculate that some, if not all, mutations have certain roles to play at different stages of HBV infection. In Figure 1D, the number of HBV SNVs positively correlated to the occurrence of HBV breakpoint was shown, however, further studies are needed to elucidate this association.

In the present study, through the systematic investigation of the relationship between HBV integration and SNV, we found that the occurrence of HBV integration events is often accompanied by an increase in SNV-positive mutations, indicating that SNV is closely related to viral integration. Additionally, the relationship between viral integration events and SNV in the TERT region is also explored in the present study, thus improving the understanding of the relationship between HBV variation and integration events.

## Supplementary Material

Supplementary Tables S1-S5

## Data Availability

All the data that support the present study have been deposited in the SRA database under the accession codes SRA335342 and SRA447498, respectively.

## Abbreviations

HBV: Hepatitis B virus
HCC: hepatocellular carcinoma
MMEJ: microhomology-mediated end joining
NHEJ: non-homologous end joining
SNV: single nucleotide variant
SRA: Sequence Read Archive
TERT: telomerase reverse transcriptase
