# Supplementary material for: Studies on the correlation between mutation and integration of HBV in hepatocellular carcinoma
Source: Biosci Rep. 2020 Aug 21;40(8):BSR20201988. doi: 10.1042/BSR20201988 (PMC7442973; doi:10.1042/BSR20201988)
Supplement: Supplementary Tables S1-S5 [file BSR-2020-1988_supp.zip › BSR-2020-1988_supp.pdf]

Table S1: Data information

Table S2: SNV of tumor samples

Table S3:Sample ratio in integrated and non-integrated samples

Table S4:Sample ratio in integrated and non-integrated samples of TERT region

Table S5:HBV breakpoints in 426 tumor samples
